# Reproductive character displacement and potential underlying drivers in a species‐rich and florally diverse lineage of tropical angiosperms (*Ruellia*; Acanthaceae)

**DOI:** 10.1002/ece3.7371

**Published:** 2021-03-16

**Authors:** Erin A. Tripp, Kyle G. Dexter, Heather B. Stone

**Affiliations:** ^1^ Department of Ecology and Evolutionary Biology University of Colorado Boulder CO USA; ^2^ Museum of Natural History University of Colorado Boulder CO USA; ^3^ School of GeoSciences University of Edinburgh Edinburgh UK; ^4^ Royal Botanic Garden Edinburgh Edinburgh UK

**Keywords:** character displacement, competition for pollinators, experimental crosses, latitudinal gradient, macroevolution, reinforcement, speciation, sympatry

## Abstract

Reproductive character displacement is a pattern whereby sympatric lineages diverge more in reproductive character morphology than allopatric lineages. This pattern has been observed in many plant species, but comparably few have sought to disentangle underlying mechanisms. Here, in a diverse lineage of Neotropical plants (*Ruellia*; Acanthaceae), we present evidence of reproductive character displacement in a macroevolutionary framework (i.e., among species) and document mechanistic underpinnings. In a series of interspecific hand pollinations in a controlled glasshouse environment, we found that crosses between species that differed more in overall flower size, particularly in style length, were significantly less likely to produce viable seeds. Further, species pairs that failed to set seed were more likely to have sympatric distributions in nature. Competition for pollinators and reinforcement to avoid costly interspecific mating could both result in these patterns and are not mutually exclusive processes. Our results add to growing evidence that reproductive character displacement contributes to exceptional floral diversity of angiosperms.

## Graphical abstract

Our study yields evidence of reproductive character displacement (RCD) in a diverse and widespread lineage of tropical plants. It is set apart from other studies in that we investigate RCD from a macroevolutionary perspective, by comparing data from experimental crosses among 33 different species pairs. We additionally generate and review evidence to help distinguish two primary drivers of RCD in animal‐pollinated plants: pollinator competition and reinforcement. Using this approach, we find evidence that more strongly supports a hypothesis of reinforcement over pollinator competition. Our study builds on prior macroevolutionary studies of RCD and underlying drivers, including landmark research in Drosophila.

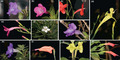

## 1 INTRODUCTION

“Endless forms most beautiful” have motivated biologists for centuries (Carroll, 2005; Darwin, 1859), and the remarkable floral diversity of angiosperms is one prime example. Floral diversity could have arisen via random drift in floral characters over time (Freckleton et al., 2002; Revell et al., 2008), with floral diversity being higher by chance in older and more diverse clades because they have had greater evolutionary history over which to accumulate floral differences. It is thought, however, that most floral diversification in angiosperms is linked to interactions with animal pollinators, given that most angiosperms (~88%) are animal pollinated—a number that rises to 94% within tropical plant communities (Ollerton et al., 2011). Pollinators have behavioral preferences for different rewards, forms, and colors of flowers, which likely contributed to a remarkable range of floral diversity (Chittka & Raine, 2006; Dudash et al., 2011; Gervasi & Schiestl, 2017; Johnson, 2010; Sargent, 2004; Smith & Kriebel, 2017; Tripp & Manos, 2008; Van der Niet & Johnson, 2012; Waser & Ollerton, 2006). Adaptation to different pollinators could readily occur in allopatry, particularly if allopatric species were exposed to different pollinators. However, given that major pollinator groups tend not to have geographically separated distributions (e.g., one does not find bats in one region, hummingbirds in another, and bees in another), much research on understanding divergence in floral form has focused on interactions with pollinators in sympatric species or within ecological communities. A key question for understanding plant evolution is whether processes at the community level are at least partly responsible for the great diversity of flowers we observe in angiosperms.

Armbruster and Muchhala (2009) outline several community‐level processes that can drive co‐occurring species to have divergent flowers. Two of these processes, (a) initial divergence in sympatry of intraspecific plant lineages due to differential pollinator utilization and (b) reinforcement of already partially reproductively isolated, previously allopatric lineages, implicate a role for pollinators in driving plant speciation. The other two processes, (c) lowering extinction risk and (d) competition for pollinators, suggest that processes which occur after postzygotic reproductive isolation is complete could drive plant species to diverge in floral form in sympatry to avoid sharing pollinators. The first process, divergence of sympatric intraspecific lineages, would suggest that sister species in plant phylogenies should be sympatric or very proximal in geographic space, but this has been repeatedly shown to generally not be the case (Armbruster & Muchhala, 2009; Tripp, 2007). Meanwhile, the last two processes are closely linked, and we consider them together under the general process of “competition for pollinators.” We therefore focus primarily on competition for pollinators and reinforcement as the two community‐level processes that are likely to generate floral diversity in angiosperm lineages. Distinguishing the relative prevalence of these two processes in angiosperm lineages is important, as the results will inform on how important pollinator interactions are for explaining not just high floral diversity, but the high species diversity of angiosperms.

When close relatives within a lineage occur in sympatry and are adapted to similar functional groups of pollinators, competition for pollinators can occur and negatively impact fitness of one or both plant species, particularly when pollinators are scarce and pollen limitation can occur (Caruso, 2000; Grossenbacher & Stanton, 2014; Muchhala et al., 2014; Sletvold et al., 2016). In such instances, selection for floral divergence in sympatry can arise, which has been documented in numerous groups of flowering plants, especially in temperate angiosperms (Sletvold et al., 2016). Competition for pollinators can thus lead to greater floral divergence in sympatry compared to allopatry, or reproductive character displacement (RCD; Grossenbacher & Stanton, 2014), which represents an important mode of ecological character displacement sensu the classical definition (MacArthur & Levins, 1967).

Meanwhile, reinforcement results from direct selection to reduce mating between sympatric lineages that already have some degree of postzygotic isolation (Coyne & Orr, 1989; Hopkins & Rausher, 2012; Hudson & Price, 2014; Matute, 2010; Wallace, 1889). Such selection can occur when postzygotic isolation is incomplete and hybrid progeny are sterile or unfit (Coyne & Orr, 1989) or when postzygotic isolation is complete and such mating represents a waste of pollen and/or prevents successful intraspecific pollen transfer by blocking the stigma (Hopkins, 2013). In contrast to selection under the competition for pollinators scenario, pollinators do not need to be scarce for reinforcing selection to occur. In angiosperms, reinforcing selection often operates on floral morphology, thus driving the evolution of morphological divergence in floral traits (Grant, 1965; Hopkins & Rausher, 2012; Kay & Schemske, 2008; Moyle et al., 2004; Silvertown et al., 2005). Reinforcement is often thought to “complete” the speciation process that begins when populations of species become isolated in allopatry but then later come into contact. While many classic studies of *Drosphila* and other animals support the concept of reinforcement, it has remained more controversial and less well‐documented in plant evolutionary biology (reviewed in Hopkins, 2013). Reinforcing selection, if common, is thought to act quickly such that natural hybrids are rarely observed.

**FIGURE 1:**
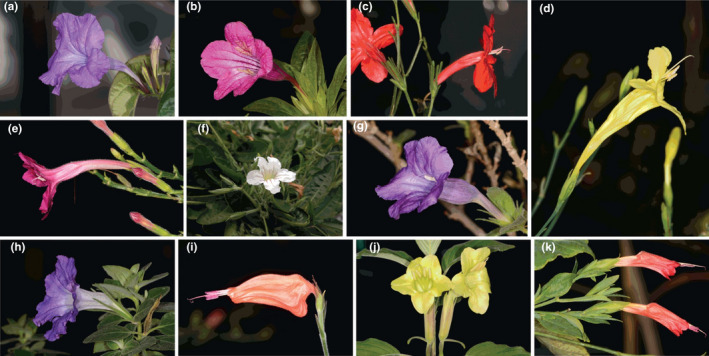
Morphological diversity of species of *Ruellia* used in this study. (a) *Ruellia breedlovei*. (b) *Ruellia macrantha*. (c) *Ruellia elegans*. (d) *Ruellia lutea*. (e) *Ruellia matudae*. (f) *Ruellia morongii*. (g) *Ruellia californica*. (h) *Ruellia hirsutoglandulosa*. (i) *Ruellia saccata*. (j) *Ruellia speciosa*. (k) *Ruellia longipedunculata*

Competition for pollinators and reinforcement are by no means mutually exclusive, and distinguishing their relative importance as drivers of RCD remains difficult (Armbruster & Muchhala, 2009; Castillo, 2017; Hopkins, 2013), despite the importance of understanding mechanisms that drive plant species divergence and floral diversification. In this study, we implement two approaches that can suggest a primary importance for one of these two processes, although as with any approach that does not experimentally manipulate natural populations, they cannot definitively rule in favor of one process or another. We apply these approaches to understand floral divergence in sympatry in a species‐rich lineage of Neotropical angiosperms (*Ruellia* L.: Wild Petunias; Figure 1). The first approach involves emphasis on the floral characters themselves that underlie RCD. Pollinators typically choose flowers based on visual and olfactory cues that signal reward (nectar and pollen, primarily) and thus divergence in these and associated characters, that is, color, tube length, and tube width, which frequently covary with reward, may signal competition for pollinators (Benitez‐Vieyra et al., 2014; Knauer & Schiestl, 2014; Ornelas et al., 2007). In contrast, under reinforcement, selection may include traits related to pollinator preference, as above, but is likely to involve additional mechanical forms of isolation or structural incompatibilities that prevent cross‐fertilization (Hopkins, 2013; Kay & Schemske, 2008). Thus, divergence in other traits not typically associated with pollinator preference, such as style length or pollen tube length, lends support to hypotheses of reinforcement over scenarios of competition for pollinators (but note it can also be indicative of other processes; Lankinen & Green, 2015).

As a second approach, artificial cross‐pollinations and resultant data on reproductive incompatibility (RI) can be employed to help further distinguish competition for pollinators from reinforcement. Under competition for pollinators (alone) as the primary driver for RCD, selection should act to reduce visitation of a given pollinator to different plant lineages (species or incipient species), but other mechanisms to prevent hybridization, such as mechanical or intrinsic isolating factors, will not necessarily manifest between plant lineages. In contrast, under reinforcement, plant lineages divergent in floral morphology, particularly those in sympatry, should also show evidence of postzygotic isolation. Hand pollinations bypass the action of pollinators and therefore offer additional means to distinguish between reinforcement and competition for pollinators. If hand pollinations between sympatric lineages and/or lineages with dissimilar flowers consistently yield nonviable offspring, reinforcement may be a primary driver of RCD. In contrast, if such hand pollinations do consistently yield viable offspring, then competition for pollinators may instead be a primary driver of RCD. As above, however, we note that competition for pollinators and reinforcement can interact, and this is not a definitive test that can exclude the role of one mechanism or the other in driving RCD.

In this study, we examine a species‐rich and florally diverse lineage of tropical angiosperms to (a) test for RCD between species pairs, which would indicate a role for community‐level processes in driving angiosperm floral diversification, and then (b) evaluate evidence in support of two different mechanisms that contribute to RCD: competition for pollinators and reinforcement. Although often investigated at a population‐level (i.e., within species), RCD nonetheless is likely to play an important role in preventing gene flow among species, that is, within a macroevolutionary context (van der Niet et al., 2006; Koski & Ashman, 2016; see also Harmon et al., 2018; Weber et al., 2018; Spriggs et al., 2019), but remains poorly studied at that evolutionary scale. We first determine if sympatric species show a pattern of RCD. We then assess which characters show the strongest pattern of RCD between species pairs. Next, we use hand pollinations in a carefully controlled glasshouse environment to test whether floral dissimilarity is correlated with RI. Finally, we assess if species pairs show greater postpollination RI when in sympatry compared to allopatry (Howard, 1993) by incorporating geographical range overlap as well as other potential effects, specifically phylogenetic relatedness. Finding that dissimilarity in floral traits (that are unlikely to be selected for by pollinators) is correlated with RI and that sympatric species cannot produce viable offspring is here taken to suggest that reinforcement may have been more important than competition for pollinators in driving RCD. The outcomes can inform future experimental research that more definitively clarifies the relative effects of these two processes and the role for RCD in plant speciation. The results from this study have implications for understanding the relative contribution of community‐level processes and RCD to floral diversification, especially given few examples are known from the tropics (but see Kay & Schemske, 2008; Muchhala et al., 2014), and serve as steps toward disentangling the underlying drivers of RCD.

## 2 MATERIALS AND METHODS

To determine whether crossing success was impacted by floral similarity, we quantified nine floral traits used in subsequent crossing trials. We generated data from five flowers per species for the following traits: length and width of the corolla tube, throat and lobes, peduncle thickness, style length, and ovary length. To determine whether crossing success was influenced by vegetative similarity (vs. floral similarity, above), we additionally quantified vegetative phenotypic divergence for these species based on five leaves per species for the following traits: leaf length, length width, petiole length, number of secondary veins, leaf apex angle, and leaf base angle. We used an Ocean Optics JAZ Spectrometer to assess floral color differences following McCarthy et al. (2017). Floral reflectance was measured three times per representative corolla at a 45˚ angle. Resulting curves were averaged and then compared across species. Overlapping spectra suggested five clear floral color bins based on curve shape, reflectance wavelength, and median peak height: purple, red, pink, yellow/green, or white (Supplementary Appendix).

**FIGURE 2:**
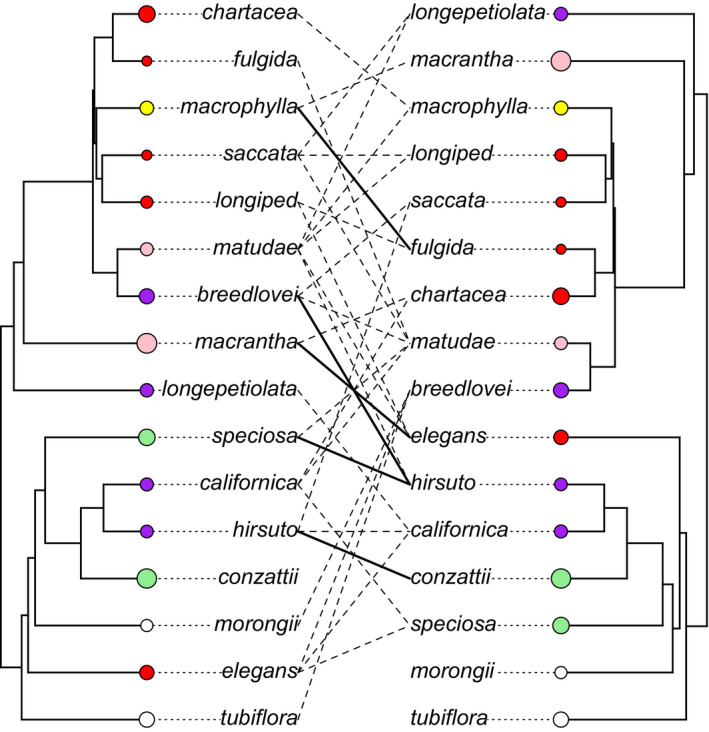
Experimental design of crossing study, showing that both allopatric and sympatric crosses were attempted within and across major clades of *Ruellia*. Lines connect species pairs for which crosses were attempted (all crosses attempted bidirectionally). Dashed lines: allopatric species pairs. Solid lines: sympatric species pairs. Circles next to species names colored according to flower color. Circles are proportional to flower size (first axis of principal component analysis in Figure S2) and depict an overall lack of phylogenetic signal for flower color or size. Phylogeny rotated around select nodes for visual clarity

To quantify the potential for hybridization, we attempted interspecific crosses for 16 species of *Ruellia* (Figure 2) growing in controlled environment glasshouses at the University of Colorado. These species were selected because they derived from the full geographical Neotropical range of *Ruellia*, with some occurring regularly in sympatry and others not. Because not all species flower at the same time, we were able to attempt crosses between a total of 33 pairs of species, in both directions (Figure 2). We focus on these pairwise comparisons when estimating drivers of crossing success, including floral similarity and geographical range overlap.

### 2.1 Hand pollinations

Hand pollinations were conducted on fresh, fully anthetic flowers by brushing mature, pollen‐coated anthers against receptive stigmas (protocol adapted from Long, 1966). This approach mirrors the direct transfer of pollen by animal pollinators in natural environments, which characterizes all species of *Ruellia*. Prior to pollinations, pollen grains were assessed visually under 10× handlens magnification for maturity, which is correlated to anther dehiscence in *Ruellia*. To ensure pollen grain viability, one of the four anthers produced by each species was removed and inspected using the lactophenol‐aniline blue stain protocol (Maneval, 1936). Stigmas were assumed to be receptive at the time of pollen maturity. Intraspecific crosses were not attempted due to limited sample size of flowers and flowering individuals. For each hand pollination, we mimicked normal pollen load by estimating the average mass produced by anthers of the maternal plant and then adjusting the dosage of pollen donated by the paternal plant accordingly. All interspecific crosses consisted of 100% interspecific pollen. Pollinations were conducted between 09:00 and 17:00. Immediately following hand pollination, receptive flowers were marked using a colored thread system to track multiple crosses on a single individual. Threads were tied loosely but securely around floral peduncles. A small pilot study conducted on flowers and leaves of six species prior to implementation of the above tracking method indicated that loose threading neither caused nor hastened tissue senescence over a two‐week period. Following visual inspection of seeds resulting from successful crosses, one to several seeds per fruit were germinated to further confirm cross success. We additionally attempted to germinate seeds from crosses deemed to be unsuccessful based on visual assessment, and none germinated.

All crosses were conducted carefully in a controlled environment in a manner that emulates direct pollen transfer by animal pollinators. Crosses were conducted reciprocally, alternating the donor/recipient status in each cross (*n* = 66 combinations in total for 33 species pairs). The total number of attempted crosses for each combination varied from 2 to 50, with 88% of all species pair combinations being attempted at least 10 times. Crosses were monitored daily until they were determined to either fail or succeed. Crosses that failed to form fruits were treated as failed crosses. Crosses that formed fruits but yielded immature and/or nonviable seeds indicate embryo failure and were treated as failed crosses. Fruits that yielded one or more mature, viable seeds based on visual inspection followed by subsequent germination trials were treated as successful crosses.

### 2.2 Molecular methods

To account for potential effects of genetic (i.e., phylogenetic) distances between species pairs, we employed the matrix from Tripp and McDade (2014a), which was constructed using three chloroplast markers plus the nuclear ITS+5.8S. We pruned this matrix to contain only taxa relevant to the present study (Figure 2). The new matrix was aligned using PhyDE (Müller et al., 2016) then analyzed using maximum likelihood implemented in RAxML v8.2 (Stamatakis, 2008). We then constructed a temporally calibrated molecular phylogeny using BEAST v1.82 (Drummond et al., 2012), with three fossil constraints (Table S1) derived from Tripp and McDade (2014b), to assess temporal divergence between species pairs. Divergence time estimation methods followed Tripp and McDade (2014b).

### 2.3 Statistical analyses

To formally test for reproductive character displacement in sympatric species pairs, we used a modified ANOSIM (analysis of similarities) approach (Clarke, 1993). First, following Coyne and Orr (1989) and Moyle et al. (2004), we classified a given species pair as sympatric if the two species overlap in some portion of their ranges. Co‐occurrence was determined through collection notes and localities of herbarium specimens and the extensive field data generated by the first author, who is a taxonomic expert on the genus. We quantified overall reproductive character similarity as the mean Euclidean distance between species in a multivariate decomposition of floral trait space, derived from a principal component analysis of the correlation matrix of the nine quantified floral characters (see Kostyun & Moyle, 2017). We also quantified the Euclidean distance between species for each individual floral character. Measures of Euclidean distance (or difference) between species for overall leaf form and individual leaf characters were calculated in the same way.

Our modified ANOSIM approach consists of ranking in decreasing order the Euclidean distances between all species pairs for a given character and then calculating how different are sympatric species pairs, compared to allopatric species pairs, for mean observed ranks. Specifically:

$$R_{\text{anosim}}=\frac{{\bar{r}}_{s}‐{\bar{r}}_{a}}{n*(n‐1)/4}$$

where ${\bar{r}}_{s}$ equals the mean rank of distances between sympatric species and ${\bar{r}}_{a}$ equals the mean rank of distances between allopatric species. The *R*_anosim_ statistic varies from 1 to −1. Values of 0 would indicate that allopatric and sympatric species pairs are no more different from each other than expected by chance. A value of 1 would indicate that sympatric species pairs are always more different in floral form for a given floral character than allopatric species pairs, while a value of −1 would indicate that allopatric species pairs are always more different. To assess whether these differences between sympatric and allopatric species pairs are significantly greater than expected by chance, we used a permutation approach where we shuffled the rows and columns of the dissimilarity matrix for a given character and obtained null expectations for the *R* value, given the pairwise values being considered (mimicking the same matrix permutation used in standard ANOSIM). This controls for nonindependence of data points involving the same species when assessing significance. For a one‐tailed test of the hypothesis that sympatric species pairs will diverge significantly more for a given trait than allopatric species pairs, we determined if the observed *R* statistic was greater than that in 95% of the permutations. In order to visualize how much more different sympatric than allopatric species pairs were for individual flower characteristics, we calculated the *F* statistic from an analysis of variance that compared the Euclidian distances for a given floral character between sympatric and allopatric species pairs.

To test whether sympatric species pairs are more likely to differ in flower color than allopatric species pairs, we conducted an initial chi‐squared analysis to assay whether these two categories of species pairs (sympatric vs. allopatric) had different ratios of species pairs with the same versus different flower colors. As this initial test showed no difference (*X* ^2^ = 0.01, *p* = 1), we did not pursue additional analyses that would have controlled for nonindependence of data points.

In order to assess drivers of interspecific crossing success, we used a generalized linear mixed model (GLMM) framework to assess how geographical range overlap and/or similarity in floral shape and color and similarity in leaf shape impacted the success of interspecific crosses (similar to Castillo, 2017). The response variable was the binomially distributed number of successes and failures for each attempted cross. We included donor and recipient species identities as random effects to control for nonindependence of crosses involving the same species and because both donor and recipient species identities have significant effects on crossing success (likelihood ratio tests of binomial GLM with species identify as fixed effect versus null model, for donor: *Χ* ^2^ = 71.2, *p* < 0.001; and recipient: *Χ* ^2^ = 95.2, *p* < 0.001). There was no relationship between crossing success in one direction versus the other (Figure S1; *r* = 0.06, *p* = 0.751), and including individual species pairs as a random effect did not improve our statistical models or change estimates of fixed effects. We also included the genetic distance between species as a fixed effect in analyses to control for this additional potential driver of crossing success. We first compared the performance of models with a single fixed effect (and the random effects) to models with only random effects using likelihood ratio tests. We then constructed a full model with all fixed and random effects and compared this full model to submodels where each fixed effect was dropped in turn, again using likelihood ratio tests. The formula for the full model is

bin(number of crosses, probability of success) ~ flower color similarity + floral shape similarity + leaf shape similarity + genetic distance + allopatry vs. sympatry + (1 | Recipient Species Identity) + (1 | Donor Species Identity)

We tested for model overdispersion using a chi‐squared test with the residual deviance and degrees of freedom. We did not attempt to test for interactions between our fixed effects due to limited sample size.

While our statistical approach accounts for nonindependence of data points due to the same species being used in multiple crosses and to variation in phylogenetic relatedness of species (following Castillo, 2017; Tobias et al., 2014), and while also correctly modeling our binomially distributed crossing success data, it is not identical to “phylogenetically corrected” approaches used in previous studies that tested the effect of sympatry versus allopatry on reproductive isolation (e.g., Coyne & Orr, 1989). In order to ensure comparability with previous studies, we conducted an additional statistical test following procedures used by Coyne and Orr (1989) and Moyle et al. (2004). Specifically, we averaged the proportion of successful crosses for all pairs of species that span a given node in our phylogeny to yield a single estimate of crossing success for each node in the phylogeny. Four of the nodes in our phylogeny were not spanned by any species pair in our study and were omitted from further analysis. Seven nodes in the phylogeny have only allopatric species pairs spanning them, while four nodes have sympatric species spanning them. We compared the mean crossing success values for nodes with only allopatric species pairs to that for nodes spanned by sympatric species pairs using a one‐tailed nonparametric Wilcoxon test.

## 3 RESULTS

**FIGURE 3:**
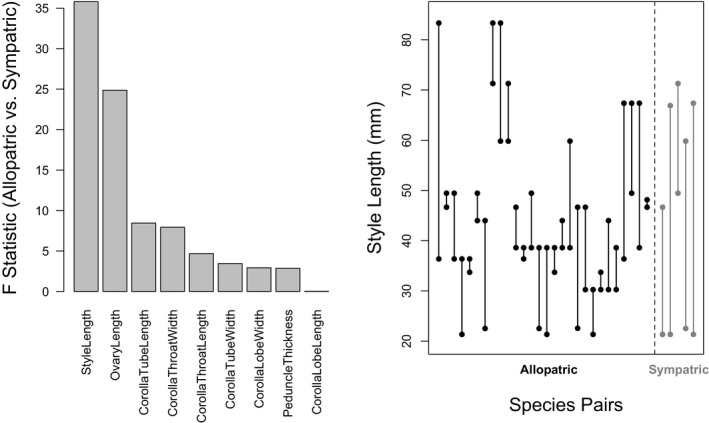
Left panel: *F*‐statistic for analyses of variance that compare interspecific distances for a given floral characteristic in sympatric versus allopatric species pairs. High values indicate that sympatric species pairs diverge more for a given floristic characteristic relative to allopatric species pairs. Low values indicate equivalent divergences. Right panel: raw style length measurements for each species pair for which a cross was attempted; each pair represented by a vertical line and end points depict style lengths for the two species. All sympatric species pairs differ by at least 21.8 mm in style length, while 23 of 28 allopatric species pairs differ by less than 21.8 mm in style length

Our results show reproductive character displacement (RCD) between sympatric species of *Ruellia* relative to allopatric species (Figure 3). Overall floral form is significantly more different between sympatric species pairs than between allopatric species pairs (*R*_anosim_ = 0.63, *p* = 0.017). Sympatric species were not significantly more different for overall leaf form (*R*_anosim_ = 0.38, *p* = 0.121). When examining individual floral characters, four of these showed a significantly greater difference between sympatric species pairs (Figure 3), with style length showing the most pronounced difference (Figure 3; *R*_anosim_ = 0.85, *p* < 0.001). In contrast, only one leaf character appears to show significantly greater differences between sympatric species pairs relative to allopatric species pairs (leaf length; *R*_anosim_ = 0.54, *p* = 0.036). If conservative Bonferroni corrections are applied to these multiple tests of significance, only style length shows a significantly greater difference between sympatric species pairs relative to allopatric species pairs.

Among 95 total crossing attempts across five sympatric species pairs, only one instance was successful and yielded mature, viable seeds (a single cross between *Ruellia conzattii* and *Ruellia hirsuto‐glandulosa*). In contrast, 63 of 730 crossing attempts between allopatric species pairs yielded mature viable seeds (Table S2). Statistical analyses using generalized linear mixed models to control for the nonindependence of data points involving the same species (following Castillo, 2017; Tobias et al., 2014) showed that occurrence in sympatry significantly reduced crossing success of species pairs (Table 1; likelihood ratio test, *Χ* ^2^ = 5.00, *df* = 1, *p* = 0.025). This result is supported by a nonparametric Wilcoxon test on a phylogenetically corrected dataset (*W* = 6, *p* = 0.032).

**TABLE 1:** Estimates of fixed effects, with standard errors, from univariate models and a multivariate model to explain crossing success in *Ruellia* (Acanthaceae)

	Univariate model estimate with *SE*	Multivariate model estimate with *SE*
Allopatry versus sympatry	−2.40 ± 1.32*	−0.78 ± 1.52
Genetic distance	−36.4 ± 18.1*	−35.3 ± 19.5
Flower color similarity	2.62 ± 0.71***	1.73 ± 0.71**
Flower shape similarity	1.23 ± 0.39***	0.92 ± 0.44*
Leaf shape similarity	0.35 ± 0.21	0.30 ± 0.29

Note

A negative coefficient for Allopatry versus Sympatry indicates reduced crossing success in sympatry relative to allopatry. Genetic distance is measured as the branch length separating two species in a maximum likelihood phylogeny. Asterisks indicate significance levels from likelihood ratio tests that (a) compare the likelihood of a model with just the single fixed effect and random effects for donor and species identity versus a null model with only random or (b) compare the likelihood of the full model with all fixed effects and random effects versus a model without the given fixed effect.

* *p* < 0.05,

** *p* < 0.01,

*** *p* < 0.001.

**FIGURE 4:**
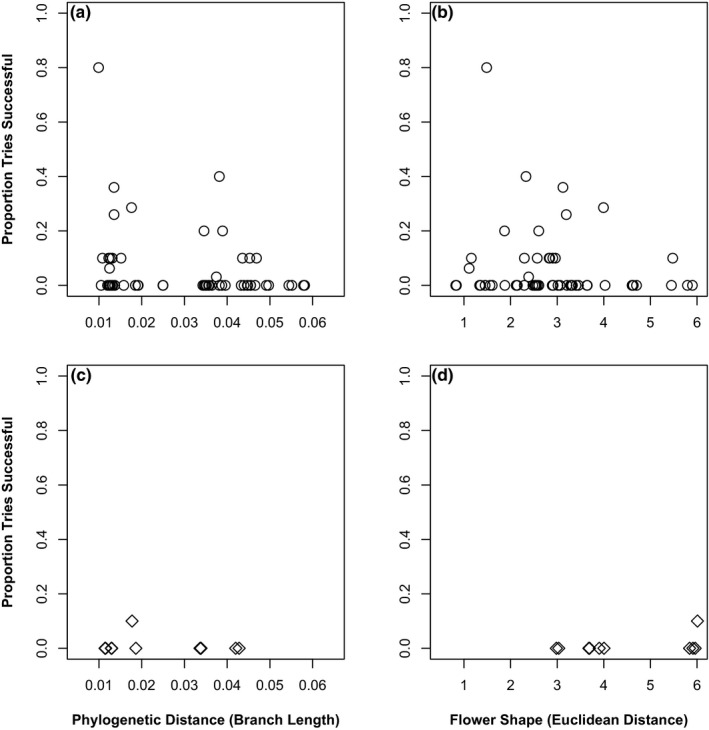
Impacts of genetic distance, measured as interspecific phylogenetic distance in a maximum likelihood phylogeny (panels a & c) and flower shape, measured as euclidean distance in a principal component decomposition of floral shape measurements (panels b & d) on crossing success. Upper panels (a & b): allopatric species pairs. Lower panels (c & d): sympatric species pairs. Floral similarity and genetic distance significantly impacted crossing success. Covariance in flower shape and geography evident in panel d (no sympatric species pairs with a flower shape distance < 3). In c and d, note that only one sympatric cross was successful. Points staggered slightly on *x*‐axis for visual clarity

Using the same generalized linear mixed model approach, we found that pairs of species with similarly shaped and similarly colored flowers had significantly higher crossing success (Table 1; Figure 4; flower color: *Χ* ^2^ = 18.94, *df* = 1, *p* < 0.001; flower shape: *Χ* ^2^ = 16.69, *df* = 1, *p* < 0.001; multivariate depiction of floral morphospace provided in Figure S2). These results are not due to a relationship between floral color and genetic distance (binomial glm, likelihood ratio test compared to null model: *Χ* ^2^ = 0.26, *df* = 1, *p* = 0.612) or floral shape and genetic distance (linear model: *F* = 0.06, *df* = 1,64, *p* = 0.813). Meanwhile, similarity in vegetative morphology did not significantly influence crossing success (Table 1; *Χ* ^2^ = 2.91, *df* = 1, *p* = 0.089). We also found that more distantly related species pairs, quantified as interspecific genetic distance in a maximum likelihood phylogeny, had significantly lower crossing success (Table 1; Figure 4; *Χ* ^2^ = 4.29, *df* = 1, *p* = 0.038). Similar results with respect to significance of fixed effects were obtained when testing the effect of time since divergence in a temporally calibrated phylogeny (Table S3). In a multivariate analysis of drivers of crossing success across all species pairs, we found the same direction for our fixed effects as in univariate analyses, but geographical range overlap of species pairs did not significantly modulate crossing success and the effect size for floral shape similarity was reduced (Table 1). An assessment of our model showed these two fixed effects to be highly correlated (*r* = 0.35) whereas none of the other fixed effects were highly correlated with each other (*r* < 0.17). If either geographical range overlap of species pairs or similarity in floral shape was removed from the model, the model performed better in explaining crossing success (Table 1; ΔAICc after removing geography = 1.3, ΔAICc after removing floral shape similarity = 0.9), but if both were removed, the model performed worse (ΔAICc = −2.5). This may be expected given the documented reproductive character displacement in sympatric species pairs.

## 4 DISCUSSION

### 4.1 The contribution of RCD to floral diversity

In plants, a pattern of increased floral divergence among sympatric compared to allopatric populations of species has now been shown for various clades (Armbruster et al., 1994; Coyne & Orr, 2004; Fishman & Wyatt, 1999; Gögler et al., 2015; Koski & Ashman, 2016; Lagomarsino & Muchhala, 2019; Muchhala & Potts, 2007; Norton et al., 2015; Whalen, 1978). In this study, we document clear patterns of reproductive character displacement (RCD) in sympatric species of a diverse lineage of Neotropical plants. Although the strongest evidence for RCD derives from studies that compare patterns of divergence among populations within a single species, the present study adds to a growing body of evidence for the pervasiveness of this pattern in plant communities (Grossenbacher & Stanton, 2014; Grossenbacher & Whittall, 2014; Koski & Ashman, 2016; Norton et al., 2015), extending the significance of the phenomenon in a macroevolutionary framework as well as in an additional lineage of tropical flowering plants. Our data in tandem with results from numerous prior studies suggest that RCD may be a major factor underlying the great floral diversity of angiosperms. The import of RCD is likely intensified among tropical latitudes given the density and diversity of plant–pollinator interactions.

### 4.2 Evidence suggestive of reinforcement

Two principle mechanisms, which are not mutually exclusive, can help explain patterns of RCD: competition for pollinators (Muchhala et al., 2014) and reinforcement (Hopkins, 2013; Kay & Schemske, 2008). Our assessment of which flower characters diverge more between sympatric species pairs than allopatric species pairs, combined with results from our artificial crossing experiments, suggest a role for reinforcement in *Ruellia*, while not excluding an additional role for competition for pollinators in driving RCD in the genus. Using hand pollinations in a controlled glasshouse environment, we found exceptionally high prezygotic isolation between sympatric species pairs: only one of 95 crossing attempts across five sympatric species pairs produced any viable seeds. In contrast, crosses between allopatric species pairs (*n* = 28 pairs) were 8× more likely to be successful (9% success rate over all crosses; 30% of crossed allopatric species pairs were successful at least once). We assessed how similar crossed species pairs were in the shape and color of their flowers, and our results suggest that divergence in floral shape, especially style length, may be one underlying mechanism that results in strong barriers to reproductive compatibility between sympatric species. Both results—strong reduction in crossability between sympatric compared to allopatric species pairs and divergence in morphological features not likely under selection by pollinators—suggest reinforcement has played a role in driving RCD in *Ruellia*. In this study, we also found that species with differently colored flowers also have greatly reduced crossing success, but sympatric species pairs were no more likely to have similar or differently colored flowers than allopatric species pairs. It may be that reinforcing selection acts more strongly on style length than it does on flower color.

Pollinators likely choose flowers based on their overall reward, shape, and color. In contrast, it seems less likely that they choose flowers based primarily on style length. Yet, we have shown that style length diverges more between sympatric species pairs relative to allopatric species pairs than any other floral trait measured (Figure 3). All sympatric species differ in style length by at least 21.8 mm, while 23 of 28 allopatric species pairs differ in style length by lesser amounts (Figure 3). If competition for pollinators, absent any involvement of reinforcing processes, were driving patterns recovered in our dataset, we would expect all or most floral traits to show divergence in sympatry instead of the highly variable divergence among characters we recovered (Figure 3). Further, style length is a key character that may underlie potential incompatibilities (Kay & Schemske, 2008), and selection on style length would serve to generate a prezygotic barrier between species where hybridization is maladaptive. Nonetheless, we recognize that reinforcing selection and competition for pollinators are not always mutually exclusive: Severe costs of pollen transfer between related species in sympatry may involve both types of processes whether or not reproductive isolation is fully complete and species boundaries remain semipermeable (Grossenbacher & Stanton, 2014; Harrison & Larson, 2014; Muchhala et al., 2014). Future research that seeks to assay more precisely where and when crosses fail would help to more fully illuminate the relevance of style length differences.

One important condition for reinforcing selection to occur is that interspecific gene flow is maladaptive and selected against (Hopkins, 2013; Kay & Schemske, 2008). We have shown that crosses between 30% of allopatric species pairs are capable of producing viable seeds. Thus, interspecific pollen transfer leading to hybridization is at least possible, as was earlier demonstrated by Long (1975). However, hybrids in *Ruellia* are rare in natural environments despite extensive geographical range overlap and contemporaneous flowering periods among numerous sets of species, suggesting it is maladaptive to be a hybrid (or that hybridization is otherwise rare to begin with). The first author (E. Tripp) has seen and studied nearly 200 of 300 Neotropical species in their native habitats (www.trippreport.com/ruellia‐pages) and in only one instance has a natural hybrid been encountered (i.e., between *Ruellia brevifolia* and *Ruellia puri*; Bolivia, *E. Tripp et al. 5971* & *5977* [COLO Herbarium]; only three putative, additional natural hybrids have been reported by other authors in the literature: Daniel, 1990 [*Ruellia amoena* & *Ruellia foetida*]; Ezcurra, 1993 [*Ruellia brevifolia* & *Ruellia longipedunculata*; *Ruellia brevicaulis* & *Ruellia coerulea*]).

An alternative explanation for RCD is a scenario where reproductive barriers between species in a given lineage are incomplete, and sympatric species that are similar in floral morphology may interbreed due to sharing of pollinators coupled with mechanical and genetic compatibility (Templeton, 1981). If gene flow between these diverged yet reproductively compatible lineages is recurrent and prolonged, such lineages may “fuse,” likely with the more fit or otherwise more abundant species in a given environment genetically swamping the less fit, less abundant species (Webb et al., 2011). Meanwhile, sympatric species that are highly dissimilar in floral form may be unable to interbreed and maintain distinct evolutionary lineages boundaries. Thus, “differential fusion” (Templeton, 1981) can yield a pattern of RCD similar to that driven by reinforcement. Although we cannot fully rule out differential fusion in this study, under such a model (and in contrast to reinforcement), natural hybrids should be commonly observed in nature between incompletely isolated lineages. However, as discussed above, natural hybrids are exceedingly rare in *Ruellia*, and thus, differential fusion is an unlikely explanation for our results.

### 4.3 Additional drivers of variation in crossing success

Consistent with studies of model systems in animal speciation biology (Coyne & Orr, 2004), we found that crossing success declines with increasing time of evolutionary divergence between species pairs. Whereas it is well established in animals that genetic distance is a significant predictor of interspecific fertility (Coyne & Orr, 1989), there has historically been less consensus in plants (Edmands, 2002; Moyle et al., 2004). Moyle et al. (2004) used comparative data from multiple species to demonstrate that increasing genetic distance strongly decreased crossability in one of the investigated study systems (*Silene*), but not in the other two lineages they examined. Similarly, using a massive dataset on species crossability in *Eucalyptus*, Larcome et al. (2015) found decreased reproductive compatibility with increasing genetic distance. Our and other studies (e.g., Brandvain et al., 2014; Moyle et al., 2004; Scopece et al., 2007) confirm a growing generality of this pattern in plants.

### 4.4 Variation in reproductive character displacement across clades and latitudes

In addition to genetic distance, at least three factors should increase opportunities for and thus the potential impacts of both reinforcing selection and competition for pollinators in driving floral divergence in sympatry: clade taxon richness, geographically wide ranging and overlapping species, and high densities of individuals within populations. In *Ruellia*, hundreds of species span one of the largest latitudinal gradients occupied by any lineage of flowering plants: ca. 80 degrees (i.e., from ~43°N near Milwaukee, Wisconsin to ~37°S in central Argentina). Over half of the ~300 New World species have broad geographical ranges (i.e., ranges that extend beyond the borders of a single country). Additionally, there exists widespread co‐occurrence of both closely related and more distantly related species in *Ruellia*, and populations often consist of tens to hundreds of individuals (McDade & Tripp, 2007; Tripp, 2007, 2010; Tripp & Luján, 2017). Species‐rich lineages in which close relatives commonly encounter one another in natural environments should, on the whole, witness greater opportunity for floral diversification via either reinforcing selection or competition for pollinators. If the above predictors are accurate, emergent properties associated with lineages such as total species number, degree of range overlap, and phylogenetic relatedness of co‐occurring species should help predict the relative frequency and importance of underlying drivers of reproductive character displacement in natural landscapes.

We expect that these emergent characteristics of lineages associated with opportunity for reinforcing selection and/or competition for pollinators should be more pronounced in tropical (compared to temperate) latitudes, where there typically exists much greater taxonomic and functional diversity of pollinators. Thus, variation in phenomena such as reinforcement across latitudes may be one mechanism contributing to latitudinal gradients in sympatric, and perhaps overall, biodiversity. *Ruellia* and other broadly ranging lineages (e.g., *Asclepias*) provide excellent systems in which to study whether and how processes including reinforcement and, presumably, competition vary with latitude in plants.

## CONFLICT OF INTEREST

None declared.

## AUTHOR CONTRIBUTIONS

**Erin A. Tripp:** Conceptualization (equal); data curation (equal); formal analysis (equal); funding acquisition (equal); investigation (equal); methodology (equal); project administration (equal); supervision (equal); writing – original draft (equal); writing – review and editing (equal). **Kyle G. Dexter:** Conceptualization (equal); data curation (equal); formal analysis (equal); funding acquisition (equal); investigation (equal); methodology (equal); project administration (equal); visualization (equal); writing – original draft (equal); writing – review and editing (equal). **Heather B. Stone:** Data curation (equal); methodology (equal); resources (equal).

## Supporting information

Supplementary Material

Figure S1

Figure S2

Supplementary Material

## Data Availability

All raw data and R scripts associated with analyses are available in Dryad (https://doi.org/10.5061/dryad.qnk98sfff).
