# Supplementary material for: Reproductive character displacement and potential underlying drivers in a species‐rich and florally diverse lineage of tropical angiosperms (Ruellia; Acanthaceae)
Source: Ecol Evol. 2021 Mar 16;11(9):4719–30. doi: 10.1002/ece3.7371 (PMC8093712; doi:10.1002/ece3.7371)
Supplement: Supplementary file 1 — Supplementary Material [file ECE3-11-4719-s003.docx]

**Supplementary Tables & Figures**

# Table S1. Fossils used as constraints for time-calibrated phylogenetic analysis and sampled outgroup genera from Ruellieae. See Tripp & McDade (2014) for further information on fossils including Fossil #. Taxa constrained refers to crown group. Additional information on morphological synapomorphies for outgroup genera can be found in Tripp et al. (2013). Zero offset, log, mean, and quantiles refer to age (in MY) parameters implemented in BEAST.

| **Fossil #** | **Taxa Constrained** | **Age** | **Zero Offset** | **Log (SD)** | **Mean** | **5% Quantile** | **95% Quantile** |
| --- | --- | --- | --- | --- | --- | --- | --- |
| NA | All taxa | None designated (used tree prior) | NA | NA | NA | NA | NA |
| 36 | 8 taxa, pseudocolpate Ruellieae excluding Trichantherinae (*Brillantaisia grotanelli, Duosperma kilimandscharium, Dyschoriste albiflora, Hygrophila costata, Mimulopsis solmsii, Petalidium ohopohense, Phaulopsis imbricata, Strobilanthes dyeriana*) | Upper Miocene (~14.55-5.3 mya) | 5.3 | 1.4 | 2.5 | 5.4 | 14.7 |
| 51 | 5 taxa, Petalidiinae + Mimulopsiinae (*Duosperma kilimandscharium, Dyschoriste albiflora, Mimulopsis solmsii, Petalidium ohopohense, Phaulopsis imbricata*) | Mio-Pliocene (~23.8-1.8 mya) | 1.8 | 1.3 | 6 | 2.1 | 23.7 |
| 43 | 3 taxa, Trichantherinae (*Bravaisia integerrima, Sanchezia speciosa, Trichanthera corymbosa*) | 10-12 mya | 10 | 0.5 | 1 | 10.4 | 12.0 |

**Table S2**. Species used in this study, number of crosses (denominator), and outcomes (number of successful crosses in numerator). Crossing success was monitored daily, and criteria for success are described in methods. An ‘x’ indicates no cross was attempted because the species did not have overlapping flowering times in the glasshouse. Taxon abbreviations as follows: br=breedlovei, ca=californica, ch=chartacea, co-conzattii, el-elegans, fu=fulgida, hi=hirsutoglandulosa, lg=longepetiolata, lp=longipedunculata, mo=morongii, mp=macrophylla, mr=macrantha, mt=matudae, sa=saccata, sp=speciosa, tu=tubiflora.

|  | **br** | **ca** | **ch** | **co** | **el** | **fu** | **hi** | **lg** | **lp** | **mo** | **mp** | **mr** | **mt** | **ss** | **sp** | **tu** |
| --- | --- | --- | --- | --- | --- | --- | --- | --- | --- | --- | --- | --- | --- | --- | --- | --- |
| **br** | x | x | x | x | 0/10 | x | 0/10 | x | x | 0/10 | x | x | 0/50 | 0/10 | x | 0/10 |
| **ca** | x | x | 0/3 | x | 0/10 | x | 0/10 | 0/10 | x | x | x | x | 0/10 | x | 0/10 | x |
| **ch** | x | 0/4 | x | x | x | x | x | x | x | x | 0/2 | 0/7 | x | x | x | x |
| **co** | x | x | x | x | x | x | 1/10 | x | x | x | x | x | x | x | x | x |
| **el** | 1/10 | 1/10 | x | x | x | x | x | x | 2/10 | x | x | 0/10 | 8/20 | x | 2/7 | x |
| **fu** | x | x | x | x | x | x | x | x | 0/10 | x | 0/10 | x | 1/10 | x | x | x |
| **hi** | 0/11 | 8/10 | x | 0/8 | x | x | x | x | x | x | x | x | 0/10 | 0/10 | 0/6 | x |
| **lg** | x | 0/10 | x | x | x | x | x | x | x | x | x | x | 0/10 | 0/10 | x | x |
| **lp** | x | x | x | x | 0/10 | 0/10 | x | x | x | x | x | x | 1/16 | 18/50 | x | x |
| **mo** | 0/9 | x | x | x | x | x | x | x | x | x | x | x | x | x | x | x |
| **mp** | x | x | 0/10 | x | x | 0/10 | x | x | x | x | x | 0/10 | 0/10 | x | x | x |
| **mr** | x | x | 1/10 | x | 0/10 | x | x | x | x | x | 0/10 | x | x | x | x | x |
| **mt** | 0/50 | 0/10 | x | x | 1/32 | 0/10 | 2/10 | 0/10 | 1/10 | x | 0/10 | x | x | 1/10 | 0/10 | x |
| **sa** | 0/10 | x | x | x | x | x | 0/10 | 1/10 | 13/50 | x | x | x | 1/10 | x | x | x |
| **sp** | x | 0/10 | x | x | 0/10 | x | 0/10 | x | x | x | x | x | 0/10 | x | x | x |
| **tu** | 0/10 | x | x | x | x | x | x | x | x | x | x | x | x | x | x | x |

**Table S3**. Estimates of fixed effects, with standard errors, from univariate models and a multivariate model to explain crossing success in *Ruellia* (Acanthaceae). A negative coefficient for Allopatry vs. Sympatry indicates reduced crossing success in sympatry. Time since divergence is measured as the branch length separating two species in a temporally-calibrated ultrametric phylogeny derived using the BEAST Software v1.82 (Drummond et al. 2012). Asterisks indicate significance levels from likelihood ratio tests that 1) compare the likelihood of a model with just the single fixed effect and random effects for donor and species identity versus a null model with only random or 2) compare the likelihood of the full model with all fixed effects and random effects versus a model without the given fixed effect [p < 0.05 *, p < 0.01 **, p < 0.001 ***].

|  | **Univariate model estimate with S.E.** | **Multivariate model estimate with S.E.** |
| --- | --- | --- |
| Allopatry vs. Sympatry | -2.40 ± 1.32* | -1.66 ± 1.68 |
| Time since Divergence | -0.40 ± 0.13*** | -0.30 ± 0.13** |
| Flower Color Similarity | 2.62 ± 0.71*** | 2.10 ± 0.80** |
| Flower Shape Similarity | 0.64 ± 0.29* | 0.51 ± 0.42 |
| Leaf Shape Similarity | 0.35 ± 0.21 | 0.06 ± 0.28 |

**Figure S1**. Crossing success rate of species in one direction versus the other (n=66 unique crossing combinations). Most crosses were not successful but those successful in one direction were generally unsuccessful in the other. Four of 33 crosses were somewhat (i.e., > 0) successful in both directions.

**Figure S2**. Multivariate depiction of flower morphospace. **Left panel**: first two principal component axes for flower shape, with samples coded according to flower color (purple, pink, red, yellow-green, and white) showing some correlation between flower color and shape. **Right panel**: correlation of flower shape variables with first two principal component axes and with one another. Greater spatial proximity of variables depicts higher correlation.
