# Supplementary material for: Reproductive character displacement and potential underlying drivers in a species‐rich and florally diverse lineage of tropical angiosperms (Ruellia; Acanthaceae)
Source: Ecol Evol. 2021 Mar 16;11(9):4719–30. doi: 10.1002/ece3.7371 (PMC8093712; doi:10.1002/ece3.7371)
Supplement: Supplementary file 4 — Supplementary Material [file ECE3-11-4719-s001.docx]

**Supplementary Appendix**: Floral colors were assigned (purple, red, pink, yellow/green, or white) using corolla reflectance measurements from an Ocean Optics JAZ-COMBO spectrophotometer. Three spectral measurements were taken per corolla per species. Pinks, purples, and reds shared a general spectral shape that resembled the letter “M”, with two major maxima and a single valley. Red individuals had a distinct spectral shape from pink and purple individuals, with the distinguishing maxima from 470-473 nm. Pinks and purples were similar in spectral shape, but with different wavelength ranges for their spectral valleys (pink at 550-555 nm, purple at 577-580 nm). Flowers with white, yellow, and green hues had flatter spectral shapes than red, purple, or pink individuals. White individuals reflected intensely from 400 nm whereas yellow and green individuals reflected from 500 nm. Herbarium voucher number (E. Tripp collections) follows taxon name (vouchers deposited at the University of Colorado Herbarium [COLO]).

Ruellia breedlovei_ET4590

Ruellia californica_ET4599

Ruellia chartacea_ET4591

Ruellia conzattii_ET4592

Ruellia elegans_ET4594

Ruellia fulgida_ET494

Ruellia hirsuto-glandulosa_ET5904?

Ruellia longipedunculata_ET4595

Ruellia longipetiolata_ET

Ruellia macrantha_ET4600

Ruellia macrophylla var. lutea_ET4598

Ruellia matudae_ET5754

Ruellia saccata_Sch-Leb60

Ruellia speciosa_ET175

Ruellia tubiflora_ET5244
